# Association between fasting blood glucose level and difficulty with chewing: the Aichi Workers’ Cohort Study

**DOI:** 10.1265/ehpm.25-00284

**Published:** 2025-12-03

**Authors:** Mohammad Hassan Hamrah, Zean Song, Youngjae Hong, Tahmina Akter, Hanson Gabriel Nuamah, Natsuko Gondo, Masaaki Matsunaga, Atsuhiko Ota, Midori Takada, Rei Otsuka, Koji Tamakoshi, Hiroshi Yatsuya

**Affiliations:** 1 Department of Public Health and Health Systems, Nagoya University Graduate School of Medicine, Nagoya, Japan; 2 Department of Public Health, Fujita Health University School of Medicine, Toyoake, Japan; 3 Department of Epidemiology of Aging, National Centre for Geriatrics and Gerontology, Obu, Aichi, Japan; 4 Department of Nursing, Nagoya University School of Health Sciences, Nagoya, Aichi, Japan

**Keywords:** Chewing difficulty, Diabetes, Epidemiological study, Japanese

## Abstract

**Background:**

Difficulty in chewing has been shown to be associated with increased mortality, geriatric syndromes, and poor activities of daily living, indicating the need for intervention. Chewing difficulties are related to tooth loss, periodontitis, dry mouth, and a number of oral health conditions. Diabetes mellitus (DM) is one of the major causes of global burden of diseases, and has been associated with poor oral health. Prospective association between oral health status and the development of diabetes has also been reported. However, relationship between glycemic control and self-reported chewing difficulty remains less explored in working-age populations. The objective of this study is to cross-sectionally explore the association between fasting blood glucose (FBG) and self-reported chewing difficulty in adults working in a Japanese worksite.

**Methods:**

Participants from the Aichi Workers’ Cohort Study who responded to the 2018 survey were included. Participants were categorized into five FBG groups (<100, 100–109, 110–125, 126–159, and ≥160 mg/dl). Multivariable odds ratios (ORs) and 95% confidence intervals (CIs) for chewing difficulty were estimated using logistic regression adjusted for age, sex, body mass index, smoking and alcohol consumption status, number of teeth, presence of periodontal disease and the number of anti-diabetic medication classes.

**Results:**

A total of 164 participants (4.2%) reported difficulty with chewing, the prevalence of which tended to increase with increasing FBG level. FBG ≥160 mg/dl was significantly and strongly associated with difficulty with chewing in the final multivariable model (multivariable OR 3.84 [95% CI 1.13–13.0]).

**Conclusions:**

A relationship between higher FBG levels and difficulty with chewing was observed, independent of potential confounding factors. However, prospective or interventional studies are needed to determine causality.

**Supplementary information:**

The online version contains supplementary material available at https://doi.org/10.1265/ehpm.25-00284.

## Graphical Abstract

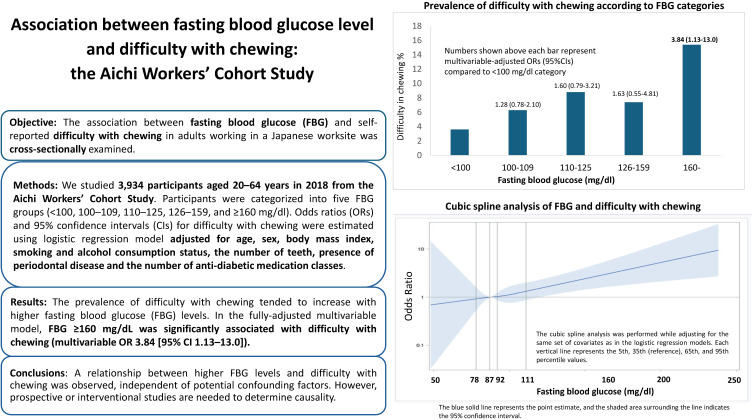

## Introduction

Oral health is an important determinant of overall systemic health, with supportive evidence linking poor oral health to higher risks for mortality, cardiovascular disease, respiratory disease, and metabolic disorders including diabetes mellitus (DM) [1–4]. Notably, DM has been suggested to cause poor oral health [5], implying a bidirectional association.

Nevertheless, fasting blood glucose (FBG) level is a critical marker of glucose control status [6, 7]. Controlling FBG levels in patients with DM reportedly reduces the risk for diabetic complications such as neuropathy, retinopathy, and nephropathy [8, 9]. One of the most common manifestations of poor oral health is difficulty with chewing [10, 11], which can have a significant impact on quality of life and nutritional status [5, 12]. Several factors, including dental diseases [13, 14], jaw disorders, and systemic conditions such as DM [15, 16] are associated with difficulty with chewing. Although previous studies, including reviews, have investigated associations between DM, poor glycemic control, and various aspects of poor oral health such as periodontitis and tooth loss [15–21]. The specific relationship between glycemic control and self-reported chewing difficulty remains less explored, particularly in working-age populations [17–19].

Previous studies have reported associations between difficulties with chewing and glycemic status, most of which have used hemoglobin A1c (HbA1c). Namely, an HbA1c level ≥6.5% was associated with self-reported difficulty with chewing [22, 23]. Similar findings were reported in a clinical study based on evaluations from a small group of participants who used a specialized device such as the Gluco Sensor GS-II (GC, Tokyo, Japan) to evaluate their chewing status [24]. However, due to the time-consuming nature of measuring chewing status using such devices, questionnaires are commonly used during health checkups.

In this study, FBG was chosen as the primary exposure due to its mandatory inclusion in Japanese worksite-based health checkups, ensuring broader applicability. Additionally, FBG can directly provide information on the presence of high glucose levels, which may capture related symptoms such as thirst. However, the present study was not designed to examine differences in the associations of FBG and HbA1c. Nevertheless, we included HbA1c results to demonstrate the consistency of the findings with those based on FBG.

In this cross-sectional study, we investigate the association among a sample of Japanese workers. Studying chewing difficulties in the working-age group is important because this population may experience unique impacts on productivity, quality of life, and long-term health outcomes due to occupational demands and lifestyle factors, differing from older adults where age-related declines predominate [20, 25, 26]. It would also be important to study this population from the prevention perspective.

Finally, previous studies did not assess other oral health variables such as the number of teeth and periodontal disease, either [22, 23]. We attempted to address some of these limitations to provide a more accurate understanding of the relationship between FBG levels and difficulty with chewing.

## Materials and methods

### Study design

The Aichi Workers’ Cohort Study is an ongoing prospective study of non-communicable diseases, including DM and cardiovascular diseases among civil servants in Japan [27]. For this cross-sectional analysis, data from individuals 20–64 years of age, who participated in the 2018 survey cycle of the cohort and provided their annual health checkup data were included (n = 5543). All participants provided informed consent. Eligibility criteria for inclusion in the current study were the availability of FBG measurement data and complete information regarding self-reported difficulty with chewing, and covariates necessary for the analysis. Namely, we excluded individuals with missing data (n = 1608) for any of the following key variables: BMI (n = 8); periodontal disease (n = 10); number of teeth (n = 132); difficulty with chewing (n = 22); FBG data (n = 1435); and those age <20 years (n = 2). As such, data from 3934 participants (2751 male, 1183 female) were analyzed. The study protocol was approved by the Ethics Review Committees of Nagoya University School of Medicine (Nagoya, Japan; Approval number: 2013-0005).

### Outcome measures

Chewing status was self-reported by responding to the question, “How is your chewing status?” Possible responses included the following: “I don’t have chewing difficulty”; “I have chewing difficulty on one side”; and “I have chewing difficulty on both sides”. In the analyses, difficulty with chewing was defined as a problem with chewing on one or both sides. Previous studies have supported the validity of this question [28, 29]. These studies evaluated chewing status by counting the number of teeth and functional tooth units (FTUs) that are closely related to chewing ability. The studies found that self-reported difficulty with chewing, the number of total and posterior teeth, and FTUs also decreased. Specifically, individuals with self-reported difficulty with chewing have approximately 3 to 4 fewer teeth than those with good chewing ability, indicating that self-reports can accurately reflect actual chewing ability [28].

### Exposure measures

Blood samples were collected from participants who fasted ≥8 hours. FBG levels were enzymatically determined using the hexokinase method with serum samples. FBG levels (mg/dl) were categorized into 5 groups: (<100, 100–109, 110–125, 126–159, and ≥160) regardless of glucose-lowering medication use. These cutoff values were selected primarily based on the established prediabetes and diabetes cutoffs. An additional cutoff of 160 mg/dl was defined primarily for exploratory purpose, but it has also been used in a previous study [30].

Hemoglobin A1c (HbA1c) levels were measured using high-performance liquid chromatography and immunoassay techniques based on National Glycohemoglobin Standardization Program (NGSP). HbA1c levels were categorized into four groups employing similar cutoffs (<5.5, 5.5–5.9, 6.0–6.4, and ≥6.5%) [31]. The analyses using HbA1c were conducted for 3160 participants with the measurement.

### Covariates

Height and weight were measured to the nearest 0.1 cm and 0.1 kg, respectively. Body mass index (BMI) was calculated as body weight (kg) divided by the square of height (m^2^).

The questionnaire items included age, sex, smoking history (never, past, current), alcohol consumption status (never, past, current), and the use of anti-diabetic medication and its names, which were classified into the number of anti-diabetic medication classes used (no medications, 1, ≥2 or insulin). The other oral conditions variables including the number of teeth and presence of periodontal disease were also self-report: Namely, we asked participants to report the number of remaining teeth and whether they had been diagnosed with periodontal disease.

### Statistical analysis

Differences in the means of continuous variables and percentages of categorical variables among the five FBG groups were tested and compared using analysis of variance and the chi-squared test, respectively. The association between FBG levels and difficulty with chewing was examined using multivariable logistic regression by estimating odds ratios (ORs) and the 95% confidence intervals (CIs). Three analytical models were used: Model 1 was adjusted for age, gender, smoking history, alcohol consumption status, and BMI; Model 2 was adjusted for the variables in Model 1 plus the number of teeth and periodontal disease; and Model 3 adjusted for the variables in Model 2 plus the number of anti-diabetic medication classes. Additionally, continuous analyses using standardized FBG (mg/dl) (i.e., divided by the standard deviation) were performed in each logistic regression model. The same analytical models were also applied to examine the association between HbA1c levels and difficulty in chewing. Since the number of subjects with HbA1c is different, a supplementary FBG-chewing difficulty analysis using a sample restricted to those with HbA1c data (n = 3160) was also performed.

Furthermore, to explore potential non-linear associations between continuous FBG and the odds of difficulty with chewing, restricted cubic spline models adjusted for the same variables (Model 3) were fitted, with knots placed at the 5th, 35th, 65th, and 95th percentiles of FBG distribution. Stratified analyses according to anti-diabetic medication use, number of teeth, and periodontal disease status were also performed using continuous FBG (standardized FBG) due to smaller number of subjects in certain strata.

All the statistical analyses were performed using SPSS version 29.0 (IBM Corp., Armonk, NY, USA) for Windows (Microsoft Corp., Redmond, WA, USA).

## Results

The mean age of the participants was 47.2 years (standard deviation: 8.9). Characteristics of the participants according to FBG group are summarized in Table 1. The mean BMI significantly increased according to the increase in FBG levels. In the high FBG group (≥160 mg/dL), there was a significantly higher prevalence of periodontal disease (38.5%), current alcohol consumption (84.6%), smoking (23.1%), and BMI ≥25 kg/m^2^ (65.4%) compared to the other FBG groups. The number of teeth did not seem to be associated with FBG groups.

**Table 1:** Participant Characteristics by FBG Groups, Aichi Workers’ Cohort Study, 2018 (N = 3934)

**Variables**	**<100** **(N = 3348)**	**100–109** **(N = 374)**	**110–125** **(N = 129)**	**126–159** **(N = 57)**	**≥160** **(N = 26)**	**P^a^**
Age, years						
mean ± SD	45.2 ± 9.9	52.2 ± 7.4	51.5 ± 7.6	52.7 ± 8.2	49.2 ± 9.7	<0.001
20–39	946 (28.3)	21 (5.6)	6 (4.7)	3 (5.3)	4 (15.4)	<0.001
40–59	2250 (67.2)	302 (80.7)	107 (82.9)	43 (75.4)	21(80.8)	
≥60	152 (4.5)	51 (13.6)	16 (12.4)	11 (19.3)	1 (3.8)	
Gender						<0.001
male	2245 (67.1)	319 (85.3)	113 (87.6)	52 (91.2)	22 (84.6)	
Smoking status						<0.001
never	2450 (73.2)	204 (54.5)	57 (44.2)	27 (47.4)	16 (61.5)	
former	591 (17.7)	116 (31.0)	46 (35.7)	20 (35.1)	4 (15.4)	
current	307 (9.2)	54 (14.4)	26 (20.2)	10 (17.5)	6 (23.1)	
Alcohol consumption status						0.011
never	728 (21.7)	63 (16.8)	16 (12.4)	12 (21.1)	4 (15.4)	
former	59 (1.8)	8 (2.1)	7 (5.4)	1 (1.8)	0 (0.0)	
current	2561 (76.5)	303 (81.0)	106 (82.2)	44 (77.2)	22 (84.6)	
Periodontal disease						0.006
present	861 (25.7)	113 (30.2)	48 (37.2)	19 (33.3)	10 (38.5)	
Number of teeth						<0.001
28 and more	2338 (69.8)	205 (54.8)	67 (51.9)	30 (52.6)	17 (65.4)	
20–27	868 (25.9)	141 (37.7)	49 (38.0)	23 (40.4)	8 (30.8)	
0–19	142 (4.2)	28 (7.5)	13 (10.1)	4 (7.0)	1 (3.8)	
Diabetes mellitus^b^						<0.001
Yes	14 (3.6)	29 (29.0)	35 (64.8)	57 (100)	26 (100)	
Anti-diabetic medication classes						<0.001
0	3340 (99.8)	362 (96.8)	110 (85.3)	37 (64.9)	12 (46.2)	
1	5 (0.1)	11 (2.9)	14 (10.9)	11 (19.3)	3 (11.5)	
≥2 or insulin	3 (0.1)	1 (0.3)	5 (3.9)	9 (15.8)	11 (42.3)	
Hba1c (%)^c^						<0.001
≥6.5	2 (0.1)	14 (4.3)	19 (16.4)	38 (69.1)	22 (84.6)	
BMI (kg/m^2^)						<0.001
mean ± SD	22.4 ± 2.9	24.5 ± 3.5	25.3 ± 4.3	26.5 ± 4.3	27.0 ± 4.0	<0.001
<21	1132 (33.8)	47 (12.6)	16 (12.4)	2 (3.5)	2 (7.7)	
21–<25	1644 (49.1)	190 (50.8)	50 (38.8)	27 (47.4)	7 (26.9)	
≥25	572 (17.1)	137 (36.6)	63 (48.8)	28 (49.1)	17 (65.4)	

The numbers of participants and their corresponding percentages are shown in the table, unless stated otherwise.

Abbreviations: FBG, fasting blood glucose; N, the number of participants; SD, standard deviation; HbA1c, hemoglobin A1c; BMI, body mass index.

^a^Analysis of variance and chi-square tests for continuous and categorical variables, respectively

^b^Diabetes mellitus was defined as fasting blood glucose (FBG) ≥126 mg/dL, HbA1c ≥6.5%, self-reported diabetes, or the use of antidiabetic medications.

^c^HbA1c data were available for 3,160 participants.

The overall prevalence of difficulty with chewing was 4.2%. The prevalence of difficulty with chewing tended to increase with higher FBG levels. Specifically, FBG ≥160 mg/dl was significantly associated with difficulty with chewing even after adjustment for the number of teeth, the presence of periodontal disease as well as the number of anti-diabetic medication classes (Model 3 OR: 3.84, 95% CI: 1.13–13.0), Continuous FBG also showed a significant positive association with chewing difficulty independent of potential confounding factors (Model 3: OR 1.13, 95% CI: 1.01–1.26) (Table 2).

**Table 2:** FBG and Chewing Difficulty: Logistic Regression, Aichi Workers’ Cohort Study (n = 3934)

**Variables**	**n/N**	**%**	**Model 1**			**Model 2**			**Model 3**		
		
			**OR**	**95% Cl**	**P**	**OR**	**95% Cl**	**P**	**OR**	**95% Cl**	**P**
FBG group (mg/dl)											
<100	121/3348	3.6	ref			ref			ref		
100–109	23/374	6.1	1.32	0.81–2.13	0.258	1.29	0.79–2.10	0.309	1.28	0.78–2.10	0.320
110–125	11/129	8.5	1.77	0.90–3.47	0.094	1.64	0.83–3.22	0.152	1.60	0.79–3.21	0.185
126–159	5/57	8.8	1.71	0.65–4.51	0.272	1.74	0.65–4.66	0.265	1.63	0.55–4.81	0.375
≥160	4/26	15.4	3.89	1.28–11.8	0.017	4.14	1.34–12.73	0.013	3.84	1.13–13.0	0.031
Continuous FBG (mg/dl)			1.16	1.04–1.29	0.006	1.16	1.04–1.29	0.004	1.13	1.01–1.26	0.031

Abbreviations: FBG, fasting blood glucose; OR, odds ratio; Cl, confidence interval; N, the number of participants; n, the number of those with chewing difficulty

Model 1 includes age, gender, smoking status, alcohol consumption statuses, and body mass index.

Model 2 includes variables in Model 1, the number of teeth, and the presence of periodontal disease.

Model 3 includes variables in Model 2, and the number of anti-diabetic medication classes.

High HbA1c group (≥6.5) was also significantly associated with difficulty with chewing independent of the number of teeth and the presence of periodontal disease (Model 2 OR: 2.26, 95% CI: 1.04–4.91), but the association weakened after adjusting for the number of anti-diabetic medication classes (Model 3 OR: 2.03, 95% CI: 0.80–5.18). However, continuous HbA1c was consistently associated with chewing difficulty (Model 3 OR: 1.16, 95% CI: 1.02–1.34) (Table 3). In a supplementary analysis, we examined the association between FBG and chewing difficulty among the 3160 participants included in the HbA1c analysis. The association was similar to the analysis using total participants (Supplementary Table 1). Also, the magnitude of the association was also similar for both measures: FBG was associated with chewing difficulty (OR = 1.17, 95% CI: 1.05–1.31), and HbA1c showed a comparable association (OR = 1.20, 95% CI: 1.07–1.35). The restricted cubic spline analysis (Supplementary Fig. 1) demonstrated a non-linear relationship between FBG and the odds of chewing difficulty, with a steeper increase in risk at higher FBG levels, particularly beyond approximately 126 mg/dL. Stratified analysis indicated that FBG was statistically significantly associated with chewing difficulty only in individuals with anti-diabetic medication (OR: 1.61, 95% CI: 1.14–2.28, p = 0.006), while no significant association was observed in those not using medication (OR: 1.10, 95% CI: 0.97–1.26, p = 0.122) (Table 4). On the contrary, FBG did not seem to be associated with chewing difficulty in those with 28 and more teeth (p for interaction = 0.019). Although the ORs of FBG with chewing difficulty were similar in those with or without periodontitis, it was statistically significant only in those without periodontitis.

**Table 3:** HbA1c and Chewing Difficulty: Logistic Regression, Aichi Workers’ Cohort Study (n = 3160)

**Variables**	**n/N**	**%**	**Model 1**			**Model 2**			**Model 3**		
		
			**OR**	**95% Cl**	**P**	**OR**	**95% Cl**	**P**	**OR**	**95% Cl**	**P**
HbA1c group (%)											
<5.5	62/1914	3.2	ref			ref			ref		
5.5–5.9	43/1003	4.3	1.09	0.73–1.64	0.656	1.08	0.71–1.63	0.699	1.16	0.77–1.75	0.459
6.0–6.4	10/150	6.7	1.34	0.64–2.78	0.427	1.39	0.66–2.92	0.373	1.40	0.68–2.87	0.355
≥6.5	10/94	10.6	2.24	1.05–4.80	0.037	2.26	1.04–4.91	0.039	1.31	0.49–3.51	0.584
Continuous HbA1c (%)			1.19	1.06–1.34	0.002	1.20	1.06–1.35	0.002	1.16	1.02–1.34	0.024

Abbreviations: HbA1c, hemoglobin A1c; OR, odds ratio; Cl, confidence interval; N, the number of participants; n, the number of those with chewing difficulty

Model 1 includes age, gender, smoking status, alcohol consumption statuses, and body mass index.

Model 2 includes variables in Model 1, the number of teeth and the presence of periodontal disease.

Model 3 includes variables in Model 2 and the number of anti-diabetic medication classes.

**Table 4:** FBG and Chewing Difficulty: Stratified by Antidiabetic Medication, Number of Teeth, and Periodontal Disease (n = 3934)

**Stratified by**	**n/N**	**Variables**	**OR**	**95% Cl**	**P**	**P for interaction**
Anti-diabetic medication use						
no	155/3850	Variables in Model 1, the number of teeth, and the presence of periodontal disease	1.10	0.97–1.26	0.122	0.067
yes	9/84		1.94	1.21–3.12	0.005	
Number of teeth						
28 and more	48/2657	Variables in Model 1, the presence of periodontal disease, and anti-diabetic medication use	0.79	0.46–1.34	0.386	0.019
20–27	101/1089		1.36	1.13–1.64	0.001	
0–19	15/188		1.40	1.03–1.89	0.027	
Periodontal disease						
absent	92/2883	Variables in Model 1, the number of teeth, and anti-diabetic medication use	1.16	1.02–1.32	0.019	0.930
present	72/1051		1.15	0.91–1.47	0.230	

Abbreviations: FBG, fasting blood glucose; OR, odds ratio; Cl, confidence interval; N, the number of participants; n, the number of those with chewing difficulty

Model 1 includes age, gender, smoking status, alcohol consumption statuses, and body mass index.

## Discussion

The current study revealed that higher FBG levels were associated with self-reported difficulty with chewing. To the best of our knowledge, this is the first study investigating the association between FBG levels and self-reported chewing status among Japanese workers. While HbA1c has been the primary marker used in most studies examining glycemic status [22, 23], we chose FBG as the primary exposure so that the findings would have broader applicability. Nevertheless, FBG and HbA1c showed similar magnitudes of association with chewing difficulties although the study was not intended to compare the two.

We found a significant and strong association between FBG levels ≥160 mg/dl and self-reported difficulty with chewing. Since the cutoff point was selected exploratorily, the present finding does not necessarily indicate the possible usefulness of the cutoff for detecting health risks. The restricted cubic spline analysis indicated a potential non-linear association with an inflection point at around 126 mg/dL; however no specific cutoff point was observed at higher levels, either.

The prevalence of chewing difficulty in the present study was 4.2%, which is considerably lower than that reported in older populations. However, the prevalence was approximately 15% among participants whose FBG was 160 mg/dL or higher, which is similar to that reported in older (65 years or older) in the JAGES population (16.7% reporting difficulty in eating) [32]. Although we did not specifically assess the types of foods with which participants experienced such difficulties, the finding remains noteworthy. Even though causality cannot be inferred from the present study, the known benefits of diabetes prevention and control should be reemphasized.

Stratified analysis according to anti-diabetic medication use revealed that the association was evident only in those taking the medication. This first suggests that individuals not taking anti-diabetic medication had lower FBG values and smaller FBG variability, resulting in lower statistical power. Second, the presence of difficulty with chewing may depend on how well FBG is controlled in individuals taking anti-diabetic medication. Third, it may merely represent that those with more severe or longer duration of diabetes could be associated with both poor glycemic control and chewing difficulty. Moreover, in those with fewer teeth, particularly in individuals with 0–19 teeth, FBG seemed to be more strongly associated with difficulty with chewing than those with 28 or more teeth independent of the presence of periodontal disease. This finding, together with the fact that FBG was not related to the number of teeth may suggest that chewing function is multifactorial and chewing difficulty may manifest under the coexistence of higher FBG levels and fewer teeth.

Previous studies have indicated a bidirectional relationship between DM and poor oral health [17–19]. Although our study is cross-sectional and cannot confirm causality, previous research has suggested possible underlying mechanisms. Poor glycemic control can lead to neuropathy, affecting sensory and motor functions necessary for effective chewing, and could cause tooth mobility and loss [20, 21]. Elevated glucose levels induce a pro-inflammatory state, which may be related to dysfunction of the muscles and joints involved in chewing [33–35]. Furthermore, high FBG is associated with dry mouth (xerostomia), which exacerbates chewing difficulties due to insufficient saliva production. High blood glucose reduces saliva flow, complicating the chewing and swallowing of dry foods [36–38]. Additionally, poor glycemic control, increased salivary glucose, and immunosuppression facilitate bacterial growth, leading to dental caries and periodontal disease, resulting in tooth loss and difficulties with chewing [39–41] although we have observed the association independent of the number of teeth and the presence of periodontal disease in the present study. As mentioned above, an association in the opposite direction is also possible. For example, chewing difficulty could lead to altered dietary habits, such as increased consumption of soft, high-carbohydrate foods, thereby worsening glycemic control [20, 36–38, 42].

The present study had several strengths and limitations. While previous studies have predominantly focused on older adults, our study examined a working-age population. Additionally, we were able to include other aspects of oral health such as the number of teeth and periodontal disease [22, 23], which strengthens the specificity of our findings. We were also able to verify the findings using HbA1c. However, this study also had some limitations, the first of which was its cross-sectional design, and the causality cannot be inferred. Second, difficulty with chewing and other oral health-related items were self-reported without clinical examination. Therefore, there may be a misclassification between self-reported chewing status and actual difficulty with chewing. However, in addition to the idea that chewing difficulty is a self-rated entity by nature, previous studies have shown the validity of self-reported chewing status [28, 29]. In addition, the validity of self-reported number of teeth and periodontal disease has been examined in previous studies [39, 40]. Self-reported tooth counts have shown strong agreement with clinically assessed counts, and self-reported periodontal disease has demonstrated acceptable validity when compared with clinical measures. Fourth, potential unmeasured confounders such as access to dental care and oral hygiene practices (e.g., frequency of brushing or flossing) may have influenced the presence or magnitude of the observed associations. Fifth, the sample size might not have been insufficient for certain categorical and stratified analyses. Finally, the study population consisted of employed adults from one worksite (local government), which may limit the generalizability of the findings. However, the participants are likely to share similar socioeconomic characteristics, reducing the possibility of substantial unmeasured confounding compared with community-based studies.

## Conclusion

In conclusion, high FBG levels were associated with higher prevalence of difficulty with chewing in a sample of the Japanese working population. Prospective studies on the relationship are warranted.

## Supplementary information

**Additional file 1:** **Supplementary Figure 1.** Cubic Spline Analysis of FBG and Chewing Difficulty Adjusted for Covariates. **Supplementary Table 1.** FBG and Chewing Difficulty: Logistic Regression, Aichi Workers’ Cohort Study (n = 3160).

## Declarations

### Data availability statements

The datasets generated and/or analyzed during the current study are not publicly available due to ethical and privacy concerns but could be available from the corresponding author on reasonable request after appropriate procedures including approval from the institutional ethics review committee.
