# Supplementary material for: Association between fasting blood glucose level and difficulty with chewing: the Aichi Workers’ Cohort Study
Source: Environ Health Prev Med. 2025 Dec 3;30:95. doi: 10.1265/ehpm.25-00284 (PMC12698362; doi:10.1265/ehpm.25-00284)
Supplement: Supplementary file 1 — Additional file 1: Supplementary Figure 1. Cubic Spline Analysis of FBG and Chewing Difficulty Adjusted for Covariates. Supplementary Table 1. FBG and Chewing Difficulty: Logistic Regression, Aichi Workers’ Cohort Study (n = 3160). [file ehpm-30-095-s001.docx]

**Supplementary Figure 1.** Cubic Spline Analysis of FBG and Chewing Difficulty Adjusted for Covariates


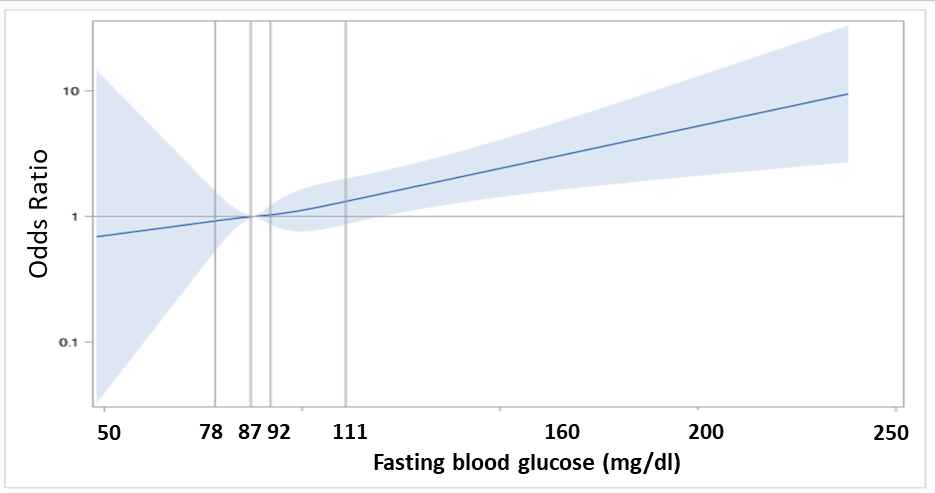


This figure shows the cubic spline model for the association between Fasting Blood Glucose (FBG) levels and self-reported chewing difficulty, adjusted for age, sex, body mass index, smoking and alcohol consumption status, number of teeth, presence of periodontal disease, and anti-diabetic medication use. Each vertical line represents the 5th, 35th (reference), 65th, and 95th percentile values. The blue solid line represents the point estimate, and the shaded area surrounding the line indicates the 95% confidence interval.

| **Supplementary Table 1.** FBG and Chewing Difficulty: Logistic Regression, Aichi Workers’ Cohort Study (n = 3160) | | | | | | | | | | | | | | | |
| --- | --- | --- | --- | --- | --- | --- | --- | --- | --- | --- | --- | --- | --- | --- | --- |
|  | |  |  | Model 1 | | |  | Model 2 | | |  | Model 3 | | | |
| Variables | n /N | % |  | OR | 95% Cl | P |  | OR | 95% Cl | P |  | OR | | 95% Cl | P |
| FBG group (mg/dl) |  |  |  |  |  |  |  |  |  |  |  |  | |  |  |
| <100 | 91/2636 | 3.5 |  | ref |  |  |  | ref |  |  |  | ref | |  |  |
| 100-109 | 17/327 | 5.2 |  | 1.27 | 0.73-2.20 | 0.388 |  | 1.21 | 0.69-2.13 | 0.309 |  | 1.20 | | 0.68-2.11 | 0.512 |
| 110-125 | 8/116 | 6.9 |  | 1.53 | 0.70-3.34 | 0.286 |  | 1.33 | 0.60-2.92 | 0.152 |  | 1.28 | | 0.57-2.87 | 0.548 |
| 126-159 | 5/55 | 9.1 |  | 2.07 | 0.77-5.52 | 0.144 |  | 2.02 | 0.74-5.50 | 0.265 |  | 1.82 | | 0.59-5.62 | 0.293 |
| ≥160 | 4/26 | 15.4 |  | 4.05 | 1.31-12.4 | 0.015 |  | 4.39 | 1.40-13.7 | 0.013 |  | 3.93 | | 1.11-13.8 | 0.034 |
| Continuous FBG (mg/dl) | |  |  | 1.09 | 1.02-1.17 | 0.007 |  | 1.10 | 1.03-1.18 | 0.005 |  | | 1.09 | 1.01-1.17 | 0.017 |
| Model 1 adjusted for: age, gender, smoking and alcohol consumption statuses, body mass index.  Model 2 adjusted for: variables in Model 1, the number of teeth, and the presence of periodontal disease.  Model 3 adjusted for: variables in Model 2, the number of anti-diabetic medication classes.  Abbreviations: FBG, fasting blood glucose; OR, odds ratio; Cl, confidence interval; N, the number of participants; n, the number of those with difficulty with chewing. | | | | | | | | | | | | | | | |
